# Testing a Dashboard Intervention for Tracking Digital Social Media Activity in Clinical Care of Individuals With Mood and Anxiety Disorders: Protocol and Design Considerations for a Pragmatic Randomized Trial

**DOI:** 10.2196/63279

**Published:** 2025-03-05

**Authors:** Brittany Nesbitt, Danielle Virgadamo, Carlos Aguirre, Matthew DeCamp, Mark Dredze, Keith Harrigian, Tenzin Lhaksampa, Jennifer M Meuchel, Aja M Meyer, Alex Walker, Ayah Zirikly, Margaret S Chisolm, Peter P Zandi, Leslie Miller

**Affiliations:** 1 Kennedy Krieger Institute Baltimore, MD United States; 2 Whiting School of Engineering Johns Hopkins University Baltimore, MD United States; 3 University of Colorado Anschutz Medical Campus Aurora, CO United States; 4 Johns Hopkins Bloomberg School of Public Health Baltimore, MD United States; 5 Johns Hopkins University School of Medicine Baltimore, MD United States; 6 Johns Hopkins All Children's Hospital St. Petersburg, FL United States

**Keywords:** digital mental health, mental health, dashboards, psychiatry, measurement-based care, electronic communication, social media, depression, anxiety, personal health information

## Abstract

**Background:**

Mood and anxiety disorders are prevalent mental health diagnoses. Numerous studies have shown that measurement-based care, which is used to monitor patient symptoms, functioning, and treatment progress and help guide clinical decisions and collaboration on treatment goals, can improve outcomes in patients with these disorders. Including digital information regarding patients’ electronic communications and social media activity is an innovative approach to augmenting measurement-based care. Recent data indicate interest and willingness from both mental health clinicians and patients to share this type of digital information in treatment sessions. However, the clinical benefit of systematically doing this has been minimally evaluated.

**Objective:**

This study aims to develop an electronic dashboard for tracking patients’ digital social activity and a protocol for a pragmatic randomized trial to test the feasibility and efficacy of using the dashboard in real-world clinical care of patients with depression or anxiety disorders.

**Methods:**

We developed a personalized electronic dashboard that tracks patients’ electronic communications and social media activity, visualizes data on these interactions through key graphics and figures, and provides a tool that can be readily integrated into routine clinical care for use by clinicians and patients during treatment sessions. We then designed a randomized trial to evaluate the feasibility and effectiveness of using the electronic dashboard in real-world care compared to treatment as usual. The trial included patients aged ≥12 years with a mood or anxiety disorder who were receiving treatment in outpatient psychiatry clinics in the Johns Hopkins Health System and the Kennedy Krieger Institute. The primary outcome includes changes in patient-rated depression symptoms. Secondary outcomes include changes in patient-rated anxiety symptoms and overall functioning. Exploratory analyses examine the impact of the intervention on measures of therapeutic alliance and the detection of clinically actionable targets.

**Results:**

We successfully developed an electronic dashboard for tracking patients’ electronic communications and social media activity, and we implemented a protocol for evaluating the feasibility and efficacy of using the dashboard in routine care for mood or anxiety disorders. The protocol was approved by the Johns Hopkins University School of Medicine Institutional Review Board. In this study, we report the technological, ethical, and pragmatic considerations in developing the dashboard and testing it in a real-world setting.

**Conclusions:**

The integration of an electronic dashboard to monitor digital social activity in mental health care treatment is novel. This study examines the feasibility and effectiveness of the dashboard and the challenges in implementing this protocol. The lessons learned from developing and implementing the study will inform ongoing discussions about the value of gathering collateral information on patients’ digital social activity and how to do so in a way that is acceptable and clinically effective.

**Trial Registration:**

ClinicalTrials.gov NCT03925038; https://clinicaltrials.gov/study/NCT03925038

**International Registered Report Identifier (IRRID):**

DERR1-10.2196/63279

## Introduction

### Background

Mood and anxiety disorders are among the most common mental health disorders in the United States, and they are associated with significant morbidity, mortality, and overall impairment in functioning [1]. These disorders are prevalent across the lifetime, with an onset often in adolescence, and evidence suggests that their rates are increasing in this age group in the United States [2]. A tragic outcome of depression and anxiety is suicide, which has increased by 30% since 2000 and is a leading cause of death in individuals aged between 10 and 34 years [3].

There are treatments for mood and anxiety disorders, but these are not always effective. In a meta-analysis of 38 studies of patients with depression and anxiety receiving outpatient mental health therapy, >40% did not show a reliable change in symptom improvement [4]. Barriers to effective treatment include patient adherence to the treatment model, session attendance, and the alignment between patients’ needs and clinicians’ therapeutic modalities [5]. There is clearly an urgent need to provide improved mental health care and develop new approaches to treat patients with mood and anxiety disorders.

Measurement-based care (MBC) is a promising but underused approach to providing more effective care for patients with mood and anxiety disorders [6]. MBC refers to the systematic use of measurement tools to monitor patient symptoms, functioning, and treatment effects in care to guide clinical decision-making and promote collaborative treatment planning [7]. A wealth of evidence shows that, when properly implemented, MBC can significantly improve patient outcomes; however, clinicians have been slow to adopt MBC in routine practice [8]. MBC traditionally entails the regular collection of patient-rated or, less frequently, clinician-rated outcome measures during treatment. A significant barrier to adopting MBC is the burden on both patients and clinicians to collect the outcome measures during busy clinical encounters [7]. The emergence of digital technologies, such as smartphones and wearables, has the potential to dramatically broaden the scope of MBC by offering new sources of collateral information that can be more readily leveraged to achieve the goals of MBC [9]. Digital information from these devices can provide valuable insights into a patient’s course of illness in real time beyond the point of care [10], and the information can be “passively” collected, which reduces the burden on patients and providers and facilitates downstream use as part of a more expansive approach to MBC [9].

A variety of information relevant to mental health can be gleaned from digital devices [9]. A particularly rich source of digital information is available from social media [10] and other forms of electronic communication, such as SMS text messages [11], collectively referred to as digital social activity in this study. Our social activity is crucial in shaping our mental health, and electronic communication and social media platforms have transformed the way people communicate and interact with one another daily. Indeed, they are typically the predominant form of communication, especially among adolescents and young adults [12,13].

A growing body of research has examined the potential for obtaining useful clinical insights into mental health from digital social activity. Multiple studies have shown that quantifiable signals derived from the language used and activity on different social media platforms are associated with and can be used to develop computational models that distinguish individuals with depression [14-17], postpartum depression [17], schizophrenia [18-20], posttraumatic stress disorder [21], and other serious mental illnesses [22,23]. Other studies have developed models that can predict clinically salient changes in the trajectories of mental illness, such as relapse leading to hospitalization in patients with psychotic disorders [22], the emergence of suicidal ideation or attempts [24,25], the occurrence of binge drinking [26], and clinical responses to antidepressant treatments [27]. Many earlier studies were limited by a reliance on self-report or inferences drawn from data available on social media platforms to establish “ground truth” about mental health end points. In addition, these studies typically analyzed data from only 1 social media platform, while individuals regularly engage with multiple platforms [28]. More recent studies have validated models using clinically documented samples with data from electronic health records [16,20], and another recent study specifically addressed the challenges of developing generalizable models based on diverse social media data sources [29]. Together, these studies suggest that it is possible to reliably monitor digital social activity for actionable collateral information, sparking interest in using such information to improve clinical care for patients with mental illnesses.

Several recent surveys have found that clinicians ask about their patients’ electronic communications and social media activity [30-32], and they believe that using this information is helpful in providing more effective treatment [32]. Both clinicians and patients have reported that they are comfortable with using and discussing social media and digital data in mental health therapy [31,33,34]. However, one study found that adult patients were less willing to share more personal data, such as their location or private communications, compared to less personal data, such as screen state (ie, phone screen on or off) and motion data [34]. Another study found that therapists were concerned about whether patients’ online posts would accurately reflect their mood, while patients did not want their use of social media and content to be the sole focus of their therapy sessions [31]. In a different study, adolescent patients at risk of suicide, whose social media activity was being monitored by their therapists, expressed concern about the balance between privacy and safety, with some reporting that they may be less likely to seek peer online support [35].

Despite the promise, it remains unclear whether systematic monitoring of digital social activity in routine care of patients with mood or anxiety disorders is acceptable and feasible. Moreover, it has not been shown whether doing so is effective in improving patient outcomes. One group has reported on the design and development of an electronic dashboard for displaying the results of computational analyses of patients’ digital social activity that can be used by clinicians and patients [36,37]; however, the use of this dashboard has not been formally tested in clinical practice. Another group has taken a simpler approach to incorporating collateral information from patients’ digital social activity and tested this approach in an unblinded randomized trial [38]. They found that integrating insights about patients’ social media use in clinical care is feasible; however, there was no significant difference in mental health outcomes between the intervention and treatment as usual (TAU) arms [38]. More research is needed to better understand the appropriate role of systematically monitoring digital social activity in routine clinical care of patients with mental illness.

### Objectives

This study was initiated to address the aforementioned need. In this study, we report on the development of an electronic dashboard for tracking patients’ digital social activity and a protocol for a pragmatic randomized trial to test the feasibility and efficacy of using the dashboard in real-world clinical care of patients with depression or anxiety disorders. We describe our guiding conceptual model and the decisions we made in designing the dashboard and protocol to test the dashboard, and we discuss the challenges we confronted and the solutions we implemented to launch the trial. The systematic monitoring of patients’ digital social activity in clinical care is not only novel and promising but also presents several important challenges. This study informs discussions about translating the promise into clinical reality and sets the stage for reporting trial results in a subsequent paper, which will ultimately contribute to the evidence base on whether monitoring digital social activity in clinical care is warranted.

## Methods

### Conceptual Model

**Figure 1:**
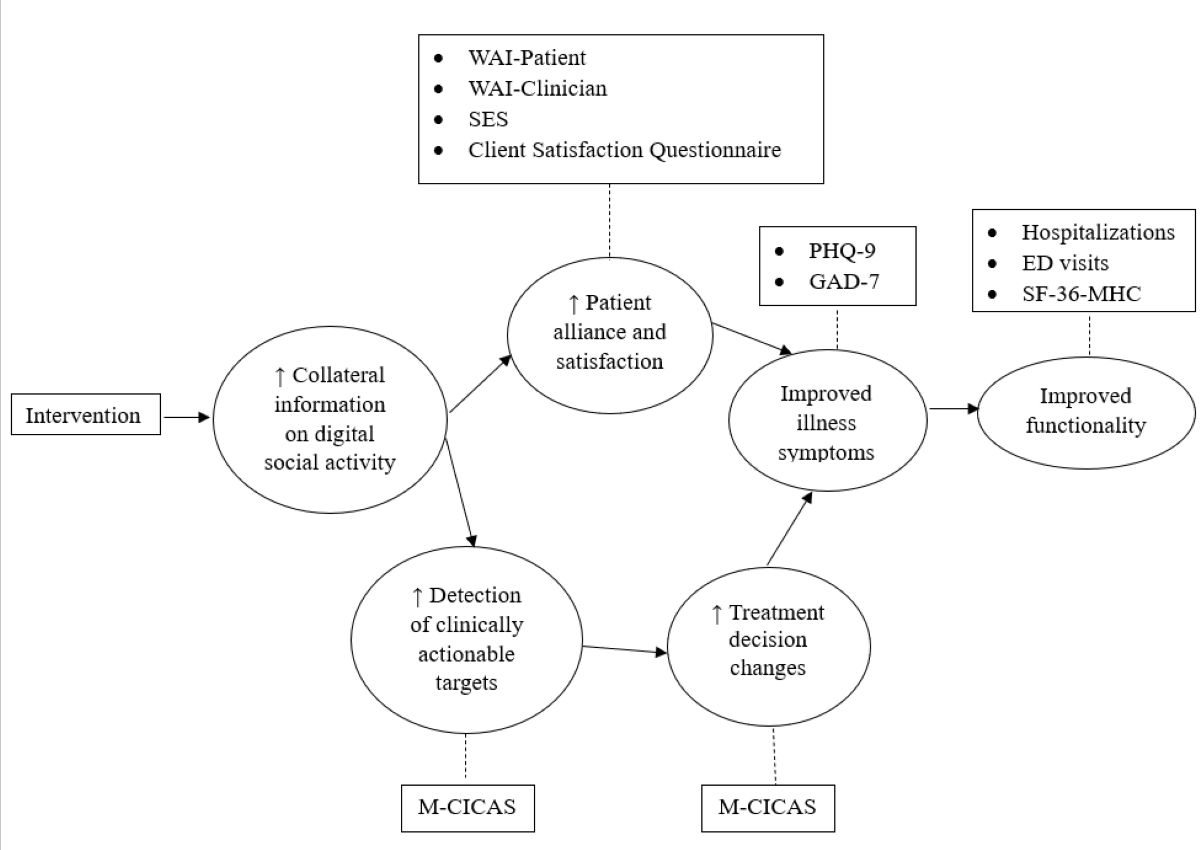
Conceptual model of the mediating pathways by which an intervention designed to monitor patients’ digital social activity may impact proximal and distal patient outcomes. ED: emergency department; GAD-7: Generalized Anxiety Disorder-7; M-CICAS: McLean Collateral Information and Clinical Actionability Scale; PHQ-9: Patient Health Questionnaire-9; SES: Session Experience Scale; SF-36 MHC: Short Form-36 Health Survey Mental Health Component; WAI: Working Alliance Inventory.

The conceptual model that guided our development and testing of a digital social media intervention in patients with depression or anxiety is shown in Figure 1.

It is grounded in the principles of MBC. We have an expansive view of MBC, which posits that systematically monitoring multiple sources of collateral information, in addition to patient- and clinician-rated outcomes measures, throughout the course of treatment can significantly improve clinical outcomes. In this case, the intervention is designed to gather collateral information about the patient’s digital social activity, which growing evidence suggests can provide important real-time insights into the patient’s mental health status. We hypothesize that such an intervention may improve downstream clinical outcomes through 2 mediating pathways. In the first pathway, routinely gathering collateral information on the patient’s digital social activity may lead to better detection of clinically actionable targets. Clinically actionable targets may include the early detection of signals that the patient’s course of illness is starting to worsen or of interpersonal struggles that may precipitate declines in the course of illness. Earlier detection of these clinically actionable targets can lead to more rapid treatment adjustments to address emerging issues before they become more intractable problems. The treatment adjustments may involve a wide variety of interventions, including changes in medication; frequency and duration of clinical visits; the focus of psychotherapy; and outreach to parents, the school, or other responsible parties in the patient’s social support network. In the second pathway, the digital social media intervention may improve the therapeutic alliance by fostering more informed and open communication between the patient and clinician, and a substantial body of evidence indicates that a strong therapeutic alliance is crucial to the success of psychiatric treatment [39]. The conceptual model hypothesizes that through these 2 mediating pathways, the intervention may improve more proximal clinical outcomes on clinical symptomatology, which, in turn, leads to improvements in more distal outcomes, such as overall functioning. It is possible that routinely collecting information about the patient’s digital social activity may lead to the false positive detection of clinically actionable targets, leading to unnecessary and perhaps harmful changes in treatment. Another possibility is that data collection may disrupt the therapeutic alliance between the patient and clinician by raising concerns over privacy and engendering distrust, which together could ultimately lead to worse clinical outcomes. These are empirical questions about the impact of the proposed intervention that need to be tested and are the focus of our randomized pragmatic trial.

### Electronic Dashboard

#### Overview

We developed an electronic dashboard to present information about our patient participants’ digital social activity for review by their treating clinician in collaboration with the participants during routine clinical encounters. The creation of each personalized dashboard involves 4 steps. First, data from different social media and electronic communication platforms used by the participants, which they have identified for release, were collected using a commercially available app called Bark. Second, the gathered data were transferred to and processed at Johns Hopkins University. Third, the data were analyzed and translated into a set of key graphs and figures that can be rendered in an electronic dashboard format. Fourth, a patient-specific dashboard was delivered to the treating clinician. For each of these components, we implemented steps to ensure the privacy and security of the data as they were gathered and processed, which are detailed in the Data Collection and Data Processing sections.

#### Data Collection

Data from the patient participants’ digital social activity were collected using Bark [40]. Bark is a commercially available app that is designed for use by parents or caregivers to monitor and manage their children’s online activity by scanning >30 social apps, web browsers, emails, and SMS text messages. It provides alerts based on algorithms trained to detect online threats, such as cyberbullying, and offers personalized insights and recommendations regarding how to address these alerts. The app works with both iOS and Android mobile devices, but differences exist in how the app accesses certain communication platforms. Apple has stricter controls to prevent third-party apps’ direct access to monitor iOS devices. Therefore, to access text messages on iOS devices, users install an app on a desktop computer, and text messages from the iOS device are “manually” transferred to the desktop via a cord connection and uploaded to Bark when the desktop is connected to the internet.

In partnership with Bark leadership, we modified the app for use in this research project by shutting down all features, including alerts, except those related to gathering data from participant activity on SMS text messages, email, and various social media platforms. As a result, the Bark app was used in our study as a data collection tool rather than an analytic tool. Textbox 1 shows the data types and platforms that are monitored by the app.

**Overview of data types and platforms monitored by Bark.**

**Data type and data sources**

- Email: AOL, Comcast, Gmail, iCloud, Outlook, and Yahoo
- Search engine: Microsoft Edge (Android), Firefox (Android), Google Chrome (Android), and Safari (iOS)
- Social media: Instagram, Twitter, Snapchat, Facebook, Pinterest, Reddit, Tumblr, and TikTok
- Text messaging: GroupMe, SMS text messages (Android and iOS), iMessage (iOS), Gmail Chat, WhatsApp, Discord, and Kik
- Miscellaneous: Google Drive (documents and comment or reply), iCloud Notes (iOS), YouTube (comment), and OneNote

#### Data Processing

Bark executes queries to their internal database at regular intervals (approximately every 30 min) that identify newly captured digital traces from all active participants in the study. These queries generated JSON-formatted data files, which were then automatically uploaded to a password-protected access point on Amazon Web Services Simple Storage Service (AWS S3). Data files were transferred through an encrypted https or transport layer security connection from AWS S3 to a remote network drive hosted at Johns Hopkins University using a scheduled job that executes hourly on a remote server. Microsoft Active Directory was used to restrict network drive access to a subset of study team members on a need-to-have basis. The study team did not provide Bark with any information about patient participants except for the unique signup codes. Data collected by Bark were not shared with guardians in the case of minor participants.

Bark does not apply any exclusion criteria to preemptively filter data uploaded to AWS S3. Instead, the study team used an intermediate processing stage to discard data that lacked natural language text, were unlikely to contain clinically relevant information (eg, promotional emails), or were of a nature that would be likely deemed too invasive by study participants. Images, videos, geolocation tags, and internet use (not including search engine queries) fell into the latter group. Text from the remaining data was passed through a toolkit, which replaced personally identifiable information (ie, phone numbers, social security numbers, zip codes, email addresses, and names) with grammatically appropriate synthetic alternatives [41].

#### Dashboard Generation

The research team developed several algorithms to analyze the electronic communication data (ie, messages) gathered via the Bark app and, in turn, created a patient participant–specific dashboard that highlighted patterns of patient electronic communication use relevant to their mental health. The choice of the information displayed on the dashboard was primarily based on previous empirical observations recorded in the literature regarding relationships among digital activity, language use, and mental health status (ie, anxiety and depression), and certain visual elements (ie, distribution of platforms used by the participant and the total number of messages sent) were included to contextualize the analyses. Some measures of use were included on the dashboard to facilitate clinician engagement with patient participants (eg, use by platform). Broadly, the type of information displayed to clinician participants about patient participants fell into the following 3 general categories: use statistics, content, and measures of content.

#### Use Statistics

The dashboard presented information regarding the frequency at which the patient participant had recently used electronic communication. To contextualize the information contained within the dashboard, digital cards located at the top of the dashboard indicated the number of messages a patient participant sent and received during the past week (with comparisons to a running weekly average), a list of platforms (eg, SMS text messages and Discord) used to send messages, and an indicator of the platform most frequently used to send messages [42,43]. To identify possible sleep disturbances or irregularities, a bar graph presented the daily distribution of messages sent during the daytime (6 AM to midnight) and late at night (midnight to 6 AM) over the past 7 days [44,45]. To provide an indication of social connectedness, a second bar graph displayed a weekly distribution of messages sent and received during the past 90 days [46]. A final bar graph displayed the weekly distribution of platforms used to send messages during the past 90 days, which was included primarily to engage participants and provide grounding to existing digital measurement tools (eg, iOS Screen Time).

#### Content

Language from messages sent by the patient participant during the past 7 days was displayed in multiple forms throughout the dashboard. First, to provide a general summary of the patient participant’s communication patterns over the previous week, a word cloud displayed the 40 most frequently used words across all digital platforms, with the size of each word dependent on its use frequency. Second, to capture recent thoughts, interests, and personal challenges, search engine queries were semantically clustered using an external knowledge base derived from Wikipedia and then listed within a table [47,48]. To highlight specific psychologically relevant themes, all messages were processed by the Linguistic Inquiry and Word Count (version 2007) toolkit [49]. The most frequently used words for a set of predefined themes were identified as psychologically relevant in previous literature [16,50-54] and representative messages for those themes were displayed. These themes included the following: positive emotion, negative emotion, anxiety, anger, sadness, family, friendship, work, health, achievement, leisure, home, money, and death. Representative messages for each theme were those that contained the highest number of thematically relevant words. Importantly, as a privacy measure, representative messages were hidden behind a pop-up window until patient participants granted their clinician verbal permission to view them. In all the aforementioned cases, common “stop words,” such as articles and pronouns, were excluded from consideration.

#### Measures of Content

Several numeric measures were also extracted from the content described earlier and visualized in the dashboard. A bar graph showed the proportion of messages sent over the previous 7 days that contained each theme from the Linguistic Inquiry and Word Count toolkit. To facilitate temporal comparisons, a heat map displayed this same measure computed weekly over the previous 90 days. A second heat map displayed the proportion of messages, aggregated weekly over the previous 90 days, containing different forms of pronouns (eg, personal vs impersonal and first person vs third person). The inclusion of the pronoun measures was based on multiple previous studies identifying correlations between the use of certain pronoun forms and mental health status [55-57].

#### Dashboard Delivery

Electronic dashboards for each patient participant were updated nightly using an automated script and stored as HTML in a password-protected relational database. Code hosted on a web server behind the Johns Hopkins University firewall was used to retrieve and display dashboards to the clinicians treating patient participants. Multiple guardrails were in place to control access to patient participant dashboards by the treating clinicians. The clinicians were allotted permission in our study’s Microsoft Active Directory group and connected to the Johns Hopkins University virtual private network to access the dashboard’s landing page. Upon reaching the dashboard’s landing page, treating clinicians were prompted to authenticate themselves using Johns Hopkins single sign-on (with 2-factor authentication). A cookie generated by the single sign-on authentication procedure was used throughout the dashboard to ensure that clinicians could only access dashboards for their own patient participants. The list of appropriate patient participants (including a participant ID and name) was shown to the clinician to facilitate navigation around the dashboard.

#### Data Privacy

We worked closely with our technology team to implement several solutions that helped preserve the privacy of the data collected through the Bark app. We developed a coding system for patients to log into the Bark app so that Bark would not have direct access to any personally identifying information about the patients who were under our care and participating in the study. In addition, we transferred and processed all the collected data to servers behind the Johns Hopkins University firewall that could only be accessed by approved study team members using Johns Hopkins University’s standard 2-factor authentication procedures. To further protect the privacy of the data collected through the app, we implemented a pipeline that minimized manual review of the data to only what was necessary to process the data and use it clinically. In addition, we instructed clinicians to review the appropriate dashboards only when they met with their patients, and at that time they could address any potential concerns that emerged from the collected data. We informed patients that the collected data would not be monitored in real time, so they were aware of the procedures.

### Randomized Trial

#### Overview

Motivated by our conceptual model, we hypothesized that providing clinicians with an electronic dashboard of their patients’ digital social activity before routine clinical encounters would aid the dialogue and exchange of relevant collateral information during those encounters and thereby improve patient outcomes. The goal was to test this hypothesis in a randomized pragmatic trial comparing patients who received care augmented by the electronic dashboard versus patients who received TAU. The primary research questions to be addressed by the trial were as follows: (1) Is the use of the electronic dashboard in routine care feasible and acceptable to both patients and their clinicians? and (2) Does the use of the electronic dashboard in routine care improve outcomes for patients with depression or anxiety disorders? The outcomes to be examined are described subsequently.

#### Study Sample

The study was carried out in outpatient psychiatry clinics across the Johns Hopkins Medical Institution and the Kennedy Krieger Institute, which is affiliated with but separate from the Johns Hopkins Medical Institution. We focused on clinics that use a collaborative care model in which patients see a psychiatrist for medication-assisted care as well as a master’s level therapist or doctoral-level psychologist for ongoing psychotherapy. Both clinicians and patients were recruited to participate in the study. We first recruited clinicians in the selected clinics to participate in the study and then sought to enroll patients on their case rolls. Patients aged ≥12 years with a mood or anxiety disorder receiving ongoing care were eligible. To be pragmatic and stay as close to the real world as possible, there were no exclusion criteria other than if the treating clinician determined the patient was not able to provide informed consent. In addition, all patients who met the inclusion criteria were eligible, regardless of their current mental health status and scores on patient or clinician rating scales.

#### Study Procedures

All patient participants downloaded the Bark app and went through the process of connecting their social media and electronic communication platforms to the app. To promote trust and participation in the study, we allowed patients to decide which platforms to connect to the Bark app. While patients retained final control of the decision, we explained to them that their most frequently used apps would provide the most relevant information. Patients were randomized within strata formed by treating clinicians in a 1:1 ratio with blocks of size 2 to either the dashboard intervention or TAU. The purpose of stratified randomization in blocks was to promote balance in the number of patient participants in the 2 treatment arms seen by each clinician, thereby reducing the impact of differences between treating clinicians on comparisons of patient outcomes between the 2 arms. For patients in the intervention arm, the electronic dashboard was delivered to the treating clinician before scheduled clinical visits. The clinician and patient collaboratively decided when and how to use the dashboard in the session during each clinical encounter. For patients in the TAU arm, the clinician did not receive a clinical dashboard for review. After the initial visit to download the Bark app, there were no more study-specific research visits. To be pragmatic and stay as close to the real-world setting as possible, patients continued to be seen by their clinician as clinically indicated. The first regularly scheduled clinic visit with their clinician after the patient consented and downloaded the Bark app was considered the study’s baseline visit. Patients were then followed through their clinical care for up to 6 months, and all study measures were completed during their regularly scheduled clinic visits.

#### Study Measures

Both the patient and clinician participants completed measures throughout the study follow-up. The measures were selected to address the primary research questions and (based on our conceptual model) to tap into potential mediating constructs. Table 1 provides the schedule of measures during the study follow-up.

**Table 1:** Schedule of participant measures.

Measures		Baseline	Every clinic visit	Every 3 months^a^
**Patient and clinician**				
	Consent	✓		
	Demographics	✓		
**Patient**				
	CSQ^b^	✓		✓
	SF-36 MHC^c^	✓		✓
	WAI-SR^d^	✓		✓
	SES^e^		✓	
	PHQ-9^f^ and GAD-7^g^		✓	
	Patient questionnaire			✓
**Clinician**				
	MFAS^h^	✓		
	M-CICAS^i^		✓	
	EDMH^j^		✓	
	WAI-SRT^k^	✓		✓
	Clinician questionnaire			✓

^a^These measures were collected every 3 months, coinciding with the nearest regularly scheduled clinic visit.

^b^CSQ: Client Satisfaction Questionnaire.

^c^SF-36 MHC: 36-Item Short Form Health Survey Mental Health Component.

^d^WAI-SR: Working Alliance Inventory-Short Revised.

^e^SES: Session Experience Scale.

^f^PHQ-9: Patient Health Questionnaire-9.

^g^GAD-7: Generalized Anxiety Disorder-7.

^h^MFAS: Monitoring and Feedback Attitudes Scale.

^i^M-CICAS: McLean Collateral Information and Clinical Actionability Scale.

^j^EDMH: Electronic Data and Mental Health.

^k^WAI-SRT: Working Alliance Inventory-Short Revised-Therapist.

#### Patient Measures

To measure proximal outcomes on mental health symptoms, participants completed the Patient Health Questionnaire-9 (PHQ-9) [58] and Generalized Anxiety Disorder-7 (GAD-7) [59] at the baseline and every subsequent clinic visit during follow-up. The PHQ-9 is a 9-item measure of current depressive symptoms ranging from 0 to 27, with higher scores representing greater levels of depression. The GAD-7 is a 7-item measure of current anxiety symptoms that ranges from 0 to 21, with higher scores representing greater levels of anxiety. In addition, to measure more distal outcomes on overall mental health–related quality of life, participants completed the Short Form-36 Health Survey Mental Health Component (SF-36 MHC) at baseline and every 3 months, coinciding with their nearest regularly scheduled clinic visit. This 13-item mental health component scoring ranges from 0 to 100, with higher scores indicating higher mental health–related quality of life [60]. We selected these measures as our primary (PHQ-9) and secondary (GAD-7 and SF-36 MHC) outcomes because they are brief, making them pragmatic to collect, and they are widely used with well-established psychometric properties for measuring important clinical outcomes that, based on the conceptual model we hypothesized, will improve with the proposed intervention.

To measure the overlapping constructs of therapeutic alliance and patient satisfaction, participants completed the Working Alliance Inventory-Short Revised (WAI-SR) and Client Satisfaction Questionnaire at baseline and every 3 months, coinciding with their nearest regularly scheduled clinic visit. The WAI-SR is a 12-item measure of the goal, task, and bond aspects of the therapeutic alliance, and each item ranges from 1 to 5, with higher scores indicating greater alliance [61]. The Client Satisfaction Questionnaire is an abbreviated 8-item measure of patient satisfaction with clinical therapy sessions, and each item ranges from 1 to 4, with higher scores reflecting greater satisfaction [62]. In addition, to further measure therapeutic alliance after every clinic visit, patients completed the Session Experience Scale. This is a 4-item scale that assesses key dimensions of effective therapeutic relationships [63]. Finally, at the end of the study follow-up, patients completed the patient questionnaire, which was a 4-item open-ended measure of the patient-clinician relationship that we developed to assess patient perception of the therapeutic relationship with their clinician and whether discussions about electronic communication had occurred and whether this was helpful.

#### Clinician Measures

At baseline, clinicians completed the Monitoring and Feedback Attitudes Scale. This is an 18-item measure of clinician attitudes toward treatment tracking and the usefulness of incorporating individualized progress measures. It is scored on a 5-point Likert scale, with higher scores indicating more positive attitudes [64]. To measure clinician perspectives on the therapeutic alliance, they completed the WAI-SR-Therapist at baseline and every 3 months, coinciding with the patient’s nearest regularly scheduled clinic visit. This is a 10-item version of the WAI-SR, and each item is rated on a 0 to 5 scale, where higher scores represent a higher alliance [65]. In addition, at every clinic visit, clinicians completed the McLean Collateral Information and Clinical Actionability Scale (M-CICAS) and the Electronic Data and Mental Health (EDMH) Questionnaire. The M-CICAS [66] collects information on the collateral sources of information reviewed, clinical actions taken, and shared decisions made between a clinician and a patient in a clinical session. The EDMH Questionnaire is a 6-item assessment of electronic communication that was completed by clinicians after sessions with patients randomized to the dashboard arm. It includes three Likert-rated questions: (1) Did review of electronic communications affect topics discussed in sessions? (2) How helpful was the review of this information to patient care? and (3) How likely is the clinician to recommend reviewing electronic communication to other clinicians? The questionnaire also includes 3 open-ended questions to gather more context about responses to the first 3 questions. Finally, at the end of the study, clinicians completed the clinician questionnaire, which is a 2-item open-ended survey to measure the use of dashboard-prompted electronic communication in discussion and the usefulness of the dashboard on patient care. We developed the EDMH Questionnaire and clinician questionnaire, but these have not been validated. They were included to gather additional qualitative data on the experience of using the dashboard in clinical care for exploratory analyses.

#### Data Management

The PHQ-9 and GAD-7 were collected as part of an MBC program that has been implemented in our clinics. As part of this program, patients completed the PHQ-9 and GAD-7 before every clinic visit through the Epic electronic health record as part of routine care. All other measures were collected remotely at the scheduled times via text with a link to questionnaire forms developed in a REDCap (Research Electronic Data Capture; Vanderbilt University) database that is maintained by approved study team members at Johns Hopkins University. These data are to be joined and processed for downstream analyses after removing all patient identifiers except for dates of service.

#### Statistical Analysis

The primary outcome of the trial is the difference in depressive symptoms measured by the PHQ-9 total score over the course of follow-up between the dashboard intervention and TAU arms. Secondary outcomes include differences in anxiety symptoms measured by the GAD-7 total score and in the more distal outcome of functioning, as measured by the SF-36 MHC. Because the PHQ-9 and GAD-7 were collected at each clinic visit and the SF-36 MHC at baseline and end of the study, there are repeated measures over time for each scale within individual patients. We plan to use random effects linear regression models to test for differences in scores on these measures between the 2 treatment arms. We will include fixed effects terms for the treatment arm as well as for time. Furthermore, we will consider including fixed effects terms for potential confounders, such as demographics (eg, age, sex, and race), and clinical factors, such as primary diagnosis and the number of clinic visits as an indicator of clinical severity. We will also include random effects for individuals to account for the correlation in outcome measures within repeated measures of the same individual as well as for clinicians, if indicated, to account for the potential clustering effects of treatment within clinicians. The primary statistical test of interest will focus on the fixed effects term for treatment, which will provide an estimate of the mean differences in the outcome measures between the 2 treatment arms, controlling for time. We will consider different functional forms of the time covariate and use the one that provides the best fit to the data on changes in score over time. Given that patients will be enrolled in the study regardless of their current mental health status and scores on these outcome measures, we consider the model in which we control for time to be the most appropriate test of differences between the treatment arms. However, we may consider examining interactions between treatment and time to see whether changes in the scores over time differ between the 2 treatment arms. We will use the conventional threshold of *P*<.05 to declare differences statistically significant.

In additional exploratory analyses, we will examine whether there are differences between the 2 treatment arms in measures of therapeutic alliance and detection of clinically actionable targets, both of which we hypothesize, based on our conceptual model, may mediate the therapeutic effect of the intervention. These analyses will provide important evidence as to whether the proposed intervention actually engages the targeted mechanisms of action. In particular, we will test whether there are differences between the 2 treatment arms on scores on the WAI-SR (which measures therapeutic alliance for both patients and clinicians) and the M-CICAS (which captures information about the detection of clinically actionable targets and whether subsequent treatment adjustments were made). For the WAI-SR score, which is a quantitative measure and was captured every 3 months, we will use the same modeling approach as statistical modeling for the primary and secondary outcomes. For the M-CICAS, which was captured after every clinic visit, we will tally the number of times issues related to the patient’s social media activity or electronic communications were discussed in the clinical encounter and the number of times treatment adjustments were made. We will again use the same modeling approach as described earlier, except that we will use random effects logistic regression models to carry out separate tests of differences in the probability of these events occurring over time between the 2 treatment arms. Furthermore, we will use *P*<.05 as the threshold to declare findings significant even though multiple tests will be carried out because these analyses will be exploratory.

Finally, we will conduct a series of analyses to assess the feasibility and acceptability of the intervention. We will use several different approaches to do this. First, we will provide descriptive statistics on how many clinicians and patients whom we approached agreed to participate and the reasons why some declined. We are particularly interested in learning whether clinicians and patients decline due to concerns about privacy in sharing personal information obtained from their social media and electronic communication activity or due to the burden of collecting and sharing this information during busy clinical encounters. Second, we will examine descriptive statistics regarding which social media and electronic communications platforms patients agreed to share to assess whether patients were selective about what information they voluntarily provided. Third, we collected a qualitative survey from clinicians to describe the main themes on whether they found systematically discussing social media and electronic communications during treatment helpful, and, if so, what they found most useful.

#### Sample Size

We estimate that the study has 80% power to detect differences in the primary outcome measure described earlier between the 2 treatment arms with a Cohen *d* effect size of at least 0.5, given the prespecified sample size of 100 and at least 3 repeated outcome measures. We selected a planned sample size of 100 to balance pragmatics and novel treatment intervention, while ensuring a large enough sample size to detect clinically meaningful differences.

### Ethical Considerations

The study protocol was approved by the Johns Hopkins University School of Medicine Institutional Review Board (#00184638). We obtained informed consent from both patients and clinicians who participated in the study, ensuring voluntary participation and understanding of objectives, procedures, and potential risks of the study. We obtained assent from patients who were aged <18 years and consent from their guardians. All personal data collected were anonymized and stored in encrypted electronic databases, accessible only to the research team members. To compensate participants for their time and effort, patients received US $100 at randomization and another US $100 at the completion of the study. Participants who shared iOS text data were compensated US $50 because of the extra work required from the participants to share these data. Therapists received US $100 for enrolling 5 patients, US $200 for enrolling 10 patients, and US $250 for enrolling ≥15 patients.

## Results

The study was initiated in April 2019, but progress was delayed due to the onset of the COVID-19 pandemic. Data collection has been completed, and the study results will be reported in a separate manuscript. The study is registered on ClinicalTrials.gov (NCT03925038).

## Discussion

### Challenges

#### Overview

The goals of this study were to create an electronic communication dashboard and evaluate its use in routine mental health treatment for patients aged ≥12 years with mood or anxiety disorders. The integration of a dashboard to monitor social media use in routine mental health care is novel and promising. With the explosion in the use of electronic communications, it is increasingly relevant to consider social media activity in the therapy setting to aid in treatment planning. However, there are important challenges that merit consideration in deciding how to best achieve this and testing whether doing so is acceptable and effective. In this study, we report the decisions we made to address these challenges in designing and testing such an intervention. The challenges generally fall into 3 categories: technological, ethical, and pragmatic.

#### Technical Challenges

The most immediate questions to tackle are technological, including how to monitor social media and electronic communications and how to extract and present the information from these data for routine use in clinical care. Regarding the first question, we decided to collaborate with a private start-up company and use the Bark app as a tool for monitoring patients’ digital social activity. Our patients typically use several social media and electronic communications platforms, and we wanted to simultaneously track as many as possible to obtain a more comprehensive picture of their digital social activity. The Bark app offers the best out-of-the-box solution that meets our needs for monitoring the widest array of platforms and working with both the iOS and Android operating systems, which are widely used by our patients. However, there are challenges in working with Bark. The app was not designed for our use case; therefore, we worked with the company to make certain modifications, to make it suitable for our purposes. To give an illustrative example, we had to shut down all alerts from the app, especially ones that promoted the product, because we did not want to be seen as endorsing it commercially. In addition, there are unavoidable challenges to making connections with the different social media and electronic communication platforms through the Bark app that are beyond its control. The iOS and Android operating systems each present their own challenges to work with, but Apple devices, in general, have stricter controls in place. Bark has implemented certain workarounds to these controls, such as for capturing SMS text messages; however, these solutions can add extra burden to patients in using the app, and it is important to learn whether this impacted their willingness to engage with the intervention. Moreover, different social media and electronic communications platforms are constantly evolving their rules and application programming interfaces for third-party access to their data, which could potentially disrupt the connections made by our patients through the Bark app. We will learn what impact, if any, this had on fidelity to the intervention throughout the course of the study. Finally, our incentives are not always aligned when working with Bark. They are a commercial enterprise and understandably motivated to focus on developing a product that will appeal to their target customers, which are parents. By contrast, we wanted to use a tool that can be seamlessly integrated into clinical care and would appeal to both our patients and their clinicians. There is clearly some but not complete overlap in our interests, and it is an open question whether working with the Bark app is sustainable beyond the life of this study.

Regarding a solution to digest and present information about the patient’s digital social activity, we decided to develop a custom electronic dashboard. Bark has developed its own algorithms to analyze social media activity data and present alerts of concerning behavior to parents. However, we chose not to use Bark’s alerts because their algorithms are proprietary, and we could not clinically validate them. As there were no other options that met our needs at the time, we decided to develop our own approach. As part of future work, we will conduct a qualitative assessment to gather stakeholder feedback on the dashboard, which can inform the next generation of development.

#### Ethical Challenges

The next set of challenges to consider are ethical challenges. A major concern is how to ethically manage personal information often exchanged on social media and other electronic communication platforms. This concern is especially heightened because we were dealing with patients as young as 12 years, and we were processing potentially sensitive information about their mental health through a third-party commercial app. We worked closely with our technology team to develop several solutions to securely gather and store data in a way that protected the privacy and confidentiality of our patients. However, these solutions raised competing concerns about how to manage scenarios where collected data might contain information that a patient was at risk of harming themselves or others. In weighing the competing concerns about privacy against responding to actionable findings revealed by the collected data, we decided to err on the side of privacy. We sought to inform patients when they joined the study that the information gathered through the Bark app was not monitored in real time and would be reviewed when they met with their clinicians. The clinicians were able to directly address any concerns about the risk of harm to the patient or others that emerged from reviewing their digital social activity during clinical sessions.

Furthermore, there was concern that social media and electronic communications may reveal information about third parties who did not consent to be in the study, such as friends, families, or acquaintances. To address this concern, we used automated procedures to anonymize the content by replacing identified names with fictitious substitutes. While no procedures for scrubbing identifiers in text are 100% effective, this step helps to minimize the chances of inadvertently revealing third-party identities. Consistent with this thinking, we also opted not to analyze data from videos or pictures. The videos and pictures are likely a rich source of information about a patient’s mental health status; however, we decided to forgo such information again in favor of caution in protecting the patient’s privacy.

#### Pragmatic Challenges

The final set of challenges to consider are pragmatic challenges. It is one thing to implement procedures that ensure the intervention is ethically sound, but it is another thing to ensure patients and providers are comfortable using those procedures. We anticipate that trust is an important factor in the adoption and success of the intervention. To respect the patient’s choice and promote trust, we chose to let participants select which social media and electronic communications platforms they connected to the Bark app. We recognize that some patients may have decided not to connect to certain platforms they regularly use because they were uncomfortable sharing their activity on these platforms, and, as a result, we may have missed important information that is highly relevant to their mental health. However, we reasoned the potential risk was worth the benefit of giving patients control over participation in the study and building trust with the intervention. We sought to explain to the patients during the log-in process the value of sharing their commonly used platforms to encourage more complete engagement. Similar reasoning motivated our decision to mask any specific content (ie, SMS text messaging) that was extracted from the social media and electronic communication platforms and displayed on the dashboard. Moreover, we wanted to give patients a measure of control over how the intervention was used and allow them to decide when clinicians could review the content. Finally, we decided to let the clinicians and patients decide collaboratively whether and when to use the dashboard during the clinical encounter. We trained the clinicians on how to interpret and use the information on the dashboard, but we let them make the decisions with their patients on how it was used in actual care. We understand that this may result in variability across clinicians on how the intervention was used, but we opted to be pragmatic and give the clinicians and patients control over the use of the intervention to foster greater buy-in.

### Limitations

In addition to the challenges detailed earlier, there are several limitations to the study that will be important to consider when interpreting the findings that come from it. First, the dashboard we developed and tested is a first-generation product, which may have important limitations in functionality. For example, it is unable to distinguish sarcasm or specific kinds of speech, such as slang, that may be important to correctly interpret social interactions. In addition, it does not include nontext forms of communication, including images or emojis, which can be rich sources of information. It is possible that the functional limitations may negatively impact the efficacy of the intervention. It will be important in future work to continue improving the dashboard to address these functional limitations and achieve the full potential of its clinical utility. Second, there is potential for selection bias in the study participants and their sharing of social media and electronic communication data. Moreover, for those who agreed to participate, their use of social media may have been influenced if they knew it was being regularly monitored. These considerations may skew the findings and limit their generalizability. Information gathered during the conduct of the study from those who do and do not agree to participate may help inform the extent to which these biases influence the results.

### Conclusions

We anticipate that this study will be an important step in advancing the use of novel tools for monitoring digital social activity in routine clinical care to improve outcomes for patients with mood or anxiety disorders. We will learn whether using such tools is acceptable and feasible and whether there is evidence that doing so provides clinical benefits. In addition, we will learn what works and what does not work when using this intervention approach, which will crucially inform how to improve upon it for real-world implementation.

## Abbreviations

AWS S3: Amazon Web Services Simple Storage Service
EDMH: Electronic Data and Mental Health
GAD-7: Generalized Anxiety Disorder-7
MBC: measurement-based care
M-CICAS: McLean Collateral Information and Clinical Actionability Scale
PHQ-9: Patient Health Questionnaire-9
REDCap: Research Electronic Data Capture
SF-36 MHC: Short Form-36 Health Survey Mental Health Component
TAU: treatment as usual
WAI-SR: Working Alliance Inventory-Short Revised

## Data Availability

The datasets generated and analyzed during this study as well as the code for the dashboard will be available from the corresponding author upon reasonable request.
